# Dance after stroke improves motor recovery in the subacute phase: A randomized controlled trial^☆^

**DOI:** 10.1016/j.heliyon.2023.e22275

**Published:** 2023-11-13

**Authors:** Anne-Violette Bruyneel, Thomas Pourchet, Aline Reinmann

**Affiliations:** a Physiotherapy Department, Geneva School of Health Sciences, HES-SO University of Applied Sciences and Arts Western Switzerland, Geneva, Switzerland

**Keywords:** Stroke, Rehabilitation, Dance, Activities, Motor, Function recovery, Satisfaction, Quality of life

## Abstract

**Purpose:**

The objective of this study was to investigate the effects of a dance program, combined with conventional treatments, on the motor recovery and quality of life of stroke survivors in comparison with conventional treatments alone.

**Materials and methods:**

A total of 16 subacute stroke survivors were randomized into two groups: a dance group (DG) and a conventional treatments group (CG). Stroke severity, cognitive abilities, and motor capacities were assessed at baseline. For six weeks, all participants underwent standard rehabilitation. However, in the DG, participants attended an additional weekly dance class. In both groups, the Mini-BESTest, Functional Independence Measure (FIM), ABC-Scale, Lower Extremity Motor Coordination Test (LEMOCOT), quadriceps strength, and Stroke-Specific Quality of Life Scale (SS-QOL) were measured at weeks 4 and 6. Nonparametric statistical tests were applied.

**Results:**

Compared to the CG, the DG significantly improved recovery of balance, coordination, and FIM after four or six weeks. No differences were observed for quadriceps strength, SS-QOL, or cognition. Participants were completely satisfied with the dance class, and no adverse effects were observed.

**Conclusions:**

This study was discontinued following the COVID-19 health crisis. However, the analysis revealed numerous beneficial effects of dance practice for subacute stroke survivors. The results contribute significantly to the advancement of artistic practices in stroke rehabilitation.

## Highlights

• A randomized controlled trial was conducted in stroke survivors.
• Compared to the control group, participants in the dance class improved balance, coordination, and functional independence.
• No effect was observed on quadriceps strength, cognition, or quality of life.
• Stroke survivors were completely satisfied with the dance classes.
• This study has been discontinued; as such, the results must be interpreted with caution.

## 1 Introduction

In 2020, the World Health Organization (WHO) published a scoping review highlighting the importance of art activities for physical, mental, and social well-being [1]. Art, based on creativity, offers imaginative experiences to both the creator and the public in order to induce an emotional response. It includes the visual arts, literature, culture, electronic arts, and performance art [1]. This last category includes active engagement in the activity, which can induce movements that require energy expenditure. Dance can therefore be considered both an artistic activity and a physical activity, allowing the positive effects on health to be combined. However, few studies have been conducted on the benefits of art activities for individuals with chronic pathologies, despite the fact that these patients exhibit symptoms that affect their physical, mental, and social health.

The annual incidence of stroke in Europe ranges between 95 and 290/100,000 (1.1 million inhabitants) [2]. This pathology, of ischemic or hemorrhagic origin, causes various motor and cognitive disorders (dementia and mild cognitive disorders in 35 % – 47 % of stroke survivors), which strongly limit daily activities and active behaviors [2,3]. Thus, cognitive-motor recovery is a major challenge in rehabilitation management to limit the long-term effects of stroke on a patient [4]. Rehabilitation is considered effective for motor recovery and the improvement of independence in daily life. However, the modalities are unclear (patient instruction, exercise type, intensity, and number of sessions), and they frequently offer exercises that target the cognitive and motor aspects separately [4]. Nevertheless, dual-task exercises appear to be more effective for balance recovery [5], and for motor learning relevant to functional movements, natural training is preferable to targeted exercises [6]. Natural activities include complex tasks with self-feedback that integrate the entire body and are diverse in terms of motor, coordination, attentional, cognitive, and relational requirements [6]. Despite the fact that motivation is essential for optimizing the effectiveness of rehabilitation, maintaining motivation during exercise can sometimes be challenging for some individuals [7]. Thus, exercises incorporating motor, cognitive, and social aspects may be highly relevant for optimizing the recovery process [8].

Dance is a global activity. It involves complex movements of the whole body through space, synchronized with music [9]. This physical activity has numerous advantages regarding the multifaceted deficits of stroke survivors. Indeed, dance class induces sensorimotor stimulation for the entire body as well as cognitive challenges and social interactions [10]. In addition, this physical activity fosters the pleasure of movement through music, group work, and the creativity of dance movement, making it a highly motivating activity in hospital settings [11]. A recent scoping review highlighted the beneficial effects of dance practice on motor and cognitive impairments, as well as the improvement in quality of life for individuals with chronic pathologies [10].

Two systematic reviews on the impact of dance classes in individuals with neurological diseases have been previously published [9,12]. Parkinson's disease has been the subject of the majority of research, with excellent results regarding symptoms and motor abilities [12]. In neurological diseases other than Parkinson's, Patterson et al. (2018) observed that dance class was highly feasible, but the conclusion regarding its positive effects was limited due to the absence of a randomized controlled trial (RCT) [9]. A recent review demonstrated the feasibility of dance classes for stroke survivors with various cognitive and motor impairments [13]. However, determining the effectiveness of this approach was not possible due to the lack of good quality studies in this context [14]. In the chronic phase, two pre-post studies demonstrated a high level of satisfaction for individuals after a stroke and an improvement in dynamic balance after dance class [15,16]. However, following a stroke, dance may have additional positive effects on balance, confidence, coordination, functional mobility, cognition, and quality of life [14]. Moreover, the potential for motor and cognitive recovery is greatest in the subacute phase [17]. Demers et al. (2015) [18] observed that dance practice was highly appreciated by patients who had a stroke within the last 6 months in a neurorehabilitation center, with very low risk. Furthermore, patients expressed a desire to continue dancing inside the hospital, as it was the most popular artistic activity prior to stroke [19]. Dancing is therefore thought to satisfy patient needs and is especially well-suited to facilitating recovery during the subacute phase with a good feasibility.

The primary aim was to perform an RCT to compare the impact of dance classes combined with conventional treatments versus conventional treatments alone on the motor recovery of individuals who had suffered a subacute stroke. Compared to control group, we hypothesized that a 6-week dance class would enhance motor recovery (balance abilities, quadriceps strength, coordination, and function). In addition, we evaluated quality of life and satisfaction. Dance practice for 6 weeks should improve quality of life and increase satisfaction in comparison with conventional treatments alone.

## 2 Materials and methods

### 2.1 Design

**Fig. 1:**
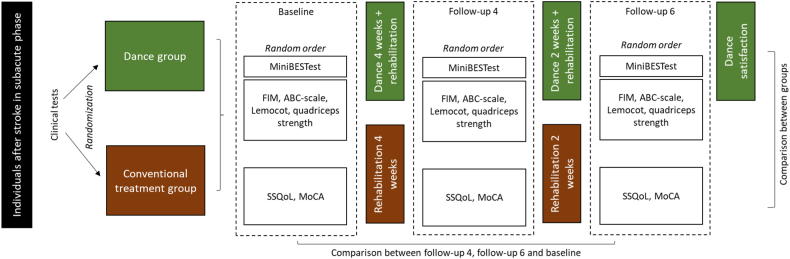
Design of the study.

This is a single-center study, parallel-arm RCT. The protocol was published previously [20]. We divided the sample into two groups: 1) dance and 2) conventional treatment. Following a baseline session before intervention, we assessed the participants after 4 weeks (follow-up 4) and 6 weeks (follow-up 6) (Fig. 1). All participants in both groups completed a 6-week standard rehabilitation program. In the dance group, participants had an additional dance class per week for 6 weeks.

### 2.2 Important changes to method after trial commencement

The trial was terminated early due to significant challenges related to the COVID health crisis. This period was marked by trial interruptions, modified care strategies, and significant team restructuring. As a result, participant recruitment was no longer possible after December 2021. The method was not changed, but we were unable to recruit the full number of participants required to calculate the sample size. This point limits the scope of the results.

### 2.3 Participants

Individuals were eligible if they were between 45 and 80 years old, had a stroke within the last 3 months (subacute phase) with medical stability, and had a sufficient cognitive level to follow the tests and dance lessons (Mini-Mental State Examination [MMSE] > 15) [21]. Additionally, the participants had to be able to sit still for 30 s and tolerate 60 min of moderate physical activity (assessed by the physiotherapist responsible for the patient at the start of the treatment). The exclusion criteria were severe hearing loss, a preexisting disease that affects walking and postural control, or major cognitive impairments. Recruitment was conducted consecutively in the neurorehabilitation department of the Institution de Lavigny (Lavigny, Vaud, Switzerland). Patients who potentially met the inclusion criteria were informed of the study. Participants were given the opportunity to sign the consent form before being enrolled in the study. This was done after at least 24 h of reflection. This study was approved by the local ethics committee (CER Vaud 2019-01467).

### 2.4 Initial clinical information

In the participants’ medical charts, the following demographic and clinical information was recorded: age, gender, type of stroke, and past medical history. Before the baseline session, the motor impairment of the lower limbs was evaluated using the Chedoke-McMaster Stroke Assessment [22]. The trunk postural control (Trunk Control Test) [23] and hemineglect (Bells Test) [24] were also evaluated.

### 2.5 Randomization

After the clinical initial tests, an investigator who did not participate in the experiment randomized the participants who met the eligibility criteria into two groups (dance and conventional treatment) by an using a computer-generated randomization tool. The permuted block randomization method was applied.

### 2.6 Interventions

All interventions were carried out at the inpatient rehabilitation center. Both groups received the same duration of face-to-face interaction with a clinician.

#### 2.6.1 Standard rehabilitation program

At the Lavigny Institution, the standard treatment after stroke consisted of daily occupational therapy sessions that included training in daily activities (45 min) and 45–60 min of physiotherapy rehabilitation. Sensory stimulation, motor training, strengthening, motor coordination, balance, and physical training were the main topics of these sessions in accordance with the objectives outlined in the initial and follow-up assessments. This rehabilitation program was conducted daily on an individual basis and two to three times per week in groups. Moreover, stroke survivors participated in neuropsychology and speech therapy sessions (three to five times per week).

#### 2.6.2 Dance class

The dance class consisted of a group session led by a dance physiotherapy instructor. To guarantee that there was a group of people (two to eight patients) for the study participants to dance with, this activity was offered to other patients not participating in the study. The frequency of one class per week was justified by earlier studies showing that this frequency was sufficient to observe positive effects in patients with motor disabilities [25,26] as well as the limited availability of resources. For safety, a physical activity specialist supported the training according to the needs of the participants.

The dance class, a combination of contemporary (including choreography) and social dance (more repetitive steps) styles, consisted of five steps accompanied by music [15,18]: 10-min warm-up, 10-min technical drills, 15-min improvisation, 15-min quick dance routine, and cool-down and feedback period [20]. Dance class included physical components (flexibility, postural control, coordination, endurance, and limb functions), social components (exercises between dancers during dance movements and a structured speaking time at the end of the dance class), and cognitive components (memory). The use of a short dance routine added a challenge for the participants to remember a sequence of moves. For the improvisation work, the dance teacher instructed the participants to follow the rhythm of the music (choice of slow, fast, or mixed music) and described a style of movement to be produced from an image while demonstrating variations on the theme (e.g., picking a fruit from a tree and placing it on the ground). The entire dance class consisted of participants interacting with one another (i.e., the whole group together or in pairs to social dance music). The dance instructor demonstrated each dance step and adjusted the complexity and intensity to each participant's ability to make it an appropriate challenge, taking into account the wide range of functional abilities that each participant had following a stroke. All dance movements can be done while sitting or standing. The dance teacher encouraged movement through verbal and motor guidance. To achieve the desired movements to the maximum of the participants' capabilities, adapted guidance was offered. However, this guidance was not systematic in order to promote pleasure in movement and prevent the participants from experiencing feelings of failure. The music had different rhythms and was chosen by both the dance teacher and the participants. The intensity of the course was regulated by rest periods between exercises.

Before the investigation began, a file was created to record the adverse events in each dance session. An adverse event was defined as a sudden event that could deteriorate the patient's health, such as a fall, a serious collision between participants, or a dance-related injury.

### 2.7 Examiners

To apply blinding conditions to the assessment, the examiners (three physiotherapists with a common training procedure) did not participate in the dance class and were unaware of the assignment of participants to each group. They were not members of the Lavigny team, but they had experience with stroke survivors.

### 2.8 Outcomes

The Mini-BESTest was the primary outcome measure used to assess balance recovery. The items evaluate the anticipatory postural adjustments, reactive postural control, sensory orientation, and dynamic gait constitute this test. Based on 14 items, each with a score between 0 and 2, where 0 is the lowest level of function and 2 is the highest, the maximum score is 28 [27]. This test has an excellent intra- and interclass reliability following a stroke (Intraclass Correlation Coefficient [ICC] > 0.99) [28].

#### ***Secondary outcomes***

All secondary outcomes were selected for their feasibility in the context of the study and for their moderate to excellent reliability (ICC > 0.60) and validity (*r* or Cronbach's alpha > 0.61) [20].

The Functional Independence Measure (FIM) instrument was applied to assess the changes in functional ability [29]. Out of the eighteen items in the FIM, thirteen evaluate physical domains and five evaluate cognitive functions. Each item is scored from 1 to 7 (“1” total dependence; “7” complete independence). The final score ranges from 18 to 126.

The balance confidence was measured using the Activities-Specific Balance Confidence Scale (ABC Scale) [30]. In 16 distinct daily activities, participants reported feeling of balance confidence. A scale of 0 % – 100 % was used to score the questionnaire (0 % representing no confidence and 100 % representing full confidence). An elevated risk of falling was indicated by a score of less than or equal to 67.

The coordination recovery was assessed using the Lower Extremity Motor Coordination Test (LEMOCOT) [31,32]. Each participant was instructed to touch the proximal and distal targets (spaced 30 cm apart) with their big toe alternately for 20 s while sitting. Each person had to keep the integrity, fluidity, and precision of their movements as well as the accuracy of their individual touches. The number of targets that were touched after a familiarization trial was counted for analysis.

The strength of the knee extensor muscle is a good indicator of the strength of other lower limb muscles following a stroke [33]. In sitting position, this muscle group was examined (feet free, knees flexed at 90°, and arms crossed) with a hand-held dynamometer placed on the distal part of the lower limb [34,35]. The participants pushed on the dynamometer for 5 s at maximum pressure. Three trials were conducted for the paretic and nonparetic sides.

The Stroke-Specific Quality of Life Scale (SS-QOL), which has been validated in French, consists of 12 subscales totaling 49 items to assess quality of life over the previous week [36]. A 5-point Likert scale with five response options was applied to each item. The final score ranged from 12 (low quality of life) to 245 (better quality of life).

To assess cognitive function, the Montreal Cognitive Assessment (MoCA) scale was used [21]. This scale is preferable to the MMSE due to its lower ceiling effect and higher internal reliability [21]. Six subsections are evaluated: language; orientation to time and space; executive functions; attention, concentration, and working memory; short-term memory; and visuospatial skills. The maximum score is 30 (better cognition), and the minimum is 0. More than 26/30 is the normal score.

A derived satisfaction scale from Patterson et al.'s (2018) study on dance therapy for people with chronic stroke was used to gauge participant satisfaction with the class [15]. On a 5-point rating scale (“1” being strongly disagreed and “5” being strongly agreed), the participants assessed nine items related to the dance program. Higher scores denoted higher levels of satisfaction.

### 2.9 Statistical methods

#### 2.9.1 Sample size

The number of subjects to be included was determined (G*Power, 2019) using the Mini-BESTest results from stroke survivors in subacute phase [37]. A difference in change of 3.5/28 points was considered clinically relevant [37]. Each group needed to have 22 participants in order to detect this difference with a standard deviation of 3.9/28, a power of 90 %, and a significance threshold of p < 0.05. Due to the COVID health crisis, this number of participants could not be reached, and the study had to be terminated prematurely.

#### 2.9.2 Statistical analysis

An independent researcher who was blinded to the group assignment used the SPSS software (SPSS Inc., Chicago, IL, USA; V.26, 2020) to perform statistical analysis.

The data treatment included the normalization of quadriceps strength with body weight and the calculation of the mean of different trials for each condition. For each group (dance vs. conventional treatment), descriptive statistics were performed including mean, standard deviation (SD), minimum and maximum for quantitative data, and frequencies for qualitative or categorical data. To evaluate the change in each parameter at follow-up 4 and follow-up 6, the change was calculated for each group (follow-up 4 or follow-up 6 value – baseline value).

Given the small sample size of each group, nonparametric tests were applied. The Mann–Whitney *U* test was used to compare the groups at baseline, follow-up 4, follow-up 6, and change values. The Wilcoxon test was performed to compare the baseline, follow-up 4, and follow-up 6 values for each group. The chi-squared test was used in the case of qualitative data. A p-value less than 0.05 was used to exclude non-significant differences.

## 3 Results

### 3.1 Participants

#### 3.1.1 Participant flow

**Fig. 2:**
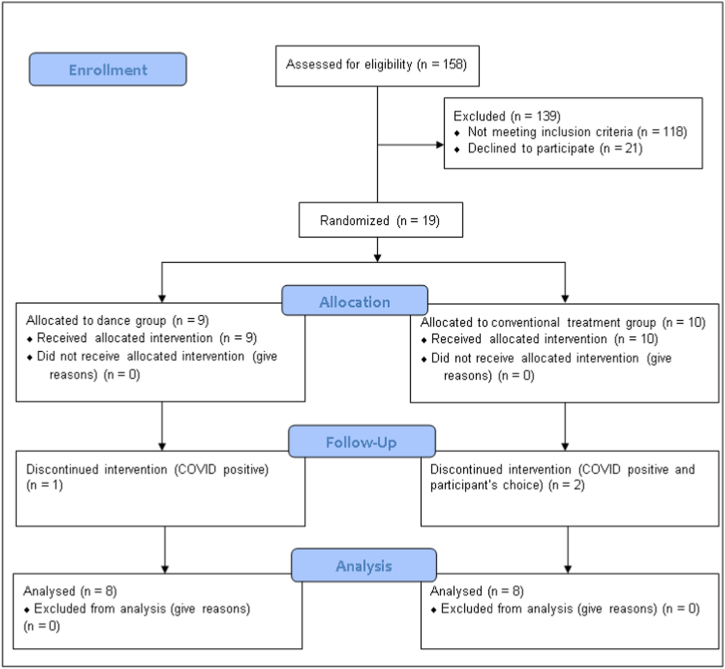
Flow chart of the study protocol.

Recruitment began in February 2020 and ended in December 2021. After 158 subacute stroke survivors were assessed for recruitment, 19 were selected (Fig. 2).

#### 3.1.2 Losses and exclusion

In total, three participants discontinued the trial: two were COVID-positive and one voluntarily withdrew. Therefore, primary and secondary outcomes were analyzed for 16 participants randomized into the dance group (N = 8) and the conventional treatment group (N = 8).

### 3.2 Baseline data

Table 1 presents the clinical and demographic characteristics. No significant differences were observed between both groups except for age (*p =* 0.041) and the Chedoke-McMaster score on the paretic foot (*p =* 0.031).

**Table 1:** Demographic variables for dance group (N = 8) and conventional treatment group group (N = 8) at baseline.

Variables	Conventional treatment group (N = 8)	Dance group (N = 8)	p value
Age (years)	64.50 ± 7.61	58.12 ± 9.15	***0.041***
Height (m)	1.75 ± 0.08	1.69 ± 0.08	0.085
Weigth (kg)	80.38 ± 18.39	74.18 ± 21.12	0.247
Sex	1 woman / 7 men	3 women / 5 men	0.564
Stroke type	1 hemorrhagic / 7 ischemic	1 hemorrhagic / 7 ischemic	0.449
Post-stroke duration (days)	24.75 ± 13.43	18.62 ± 6.07	0.146
Hemiparetic side	8 left	3 right / 5 left	0.200
Mini-Mental State Examination (score/30)	24.00 ± 4.27	25.85 ± 3.09	0.199
Chedoke-Mc Master paretic leg (score/7)	3.00 ± 1.60	4.25 ± 2.18	0.065
Chedoke-Mc Master paretic foot (score/7)	2.37 ± 1.51	4.5 ± 2.27	***0.031***
Trunk control test (score/100)	76.25 ± 30.43	90.50 ± 22.09	0.167
Bells test: - Score/35 - Duration (min)	30.87 ± 5.35 3 min 29 ± 1 min 60	32.16 ± 3.82 3 min 33 ± 1 min 18	0.500 0.437

The *p* value in italic and bold represents a significant result (*p < 0.050*).

### 3.3 Outcomes

#### 3.3.1 Adherence in dance group

All participants in the dance group attended all sessions during their stay at the rehabilitation center.

### 3.4 Results for primary outcome: Mini-BESTest

After 4 and 6 weeks of dance intervention, the Mini-BESTest values increased significantly for the dance group (*p* ≤ 0.022), whereas the conventional treatment group had no significant results for either follow-up (Table 2).

**Table 2:** Results at baseline, follow-up 4 (F4) and follow-up 6 (F6) for MiniBESTest, FIM and ABC scale.

	Group	Baseline	F4	F6	Delta (F4 – baseline)	Delta (F6 – baseline)	*p value* F4-baseline	*p value* F6 - baseline
Mini-BESTest	Dance	14.12 ± 10.09	20.00 ± 9.53	21.37 ± 8.79	5.87 ± 5.19	7.25 ± 7.26	***0.022***	***0.014***
	Conventional treatment	7.31 ± 9.21	11.87 ± 11.20	12.12 ± 11.53	4.56 ± 5.73	4.81 ± 6.31	0.059	0.059
	*p value*	0.101	0.062	***0.032***	0.426	0.268		
*FIM (score/245)*	Dance	90.12 ± 19.06	109.75 ± 11.90	112.75 ± 10.99	19.62 ± 13.30	22.62 ± 14.96	***0.022***	***0.022***
	Conventional treatment	77.20 ± 32.58	88.16 ± 34.23	88.12 ± 30.17	7.20 ± 5.17	7.20 ± 5.17	0.100	0.100
	*p value*	0.278	0.068	***0.012***	***0.018***	***0.018***		
*ABC scale (score/100)*	Dance	51.67 ± 24.76	70.83 ± 22.64	67.78 ± 20.64	19.17 ± 15.20	16.11 ± 26.26	***0.007***	0.078
	Conventional treatment	45.56 ± 31.26	57.78 ± 30.56	53.06 ± 24.75	12.22 ± 16.02	7.50 ± 22.18	0.078	0.250
	*p value*	0.337	0.245	0.619	0.113	0.246		

The *p* value in italic and bold represents a significant result (*p < 0.050*).

**Fig. 3:**
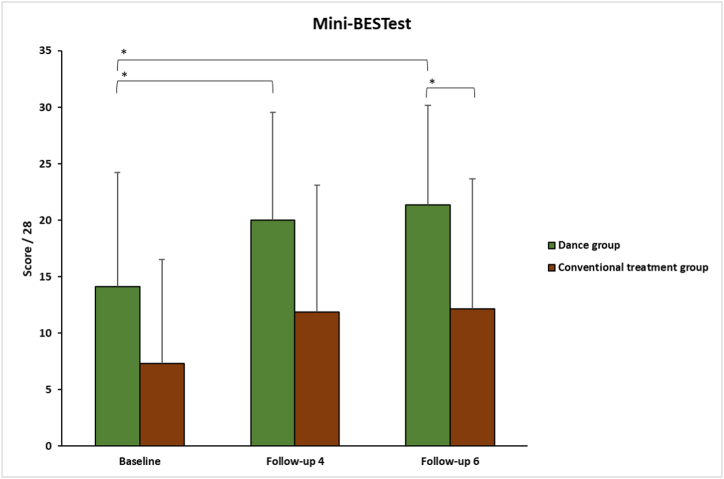
Mini-BESTest results for dance group (green) and conventional treatment group (brown). The significant difference is represented by “*”.

Between both groups, there were no significant differences at baseline and follow-up 4. At follow-up 6, participants in the dance group had a significantly higher score than the conventional treatment group (*p =* 0.032; Fig. 3), but the comparison between delta values was still not significant.

### 3.5 Results for secondary outcomes

After 4 weeks, participants in the dance group showed a significant improvement (p ≤ 0.022; Table 2), while the conventional treatment group showed no significant change in the FIM and ABC scales. The comparison between groups revealed that the dance group had greater values than the conventional treatment group for only follow-up 6 (*p =* 0.012). Using the ABC Scale to measure balance confidence, no differences were found between the groups.

**Fig. 4:**
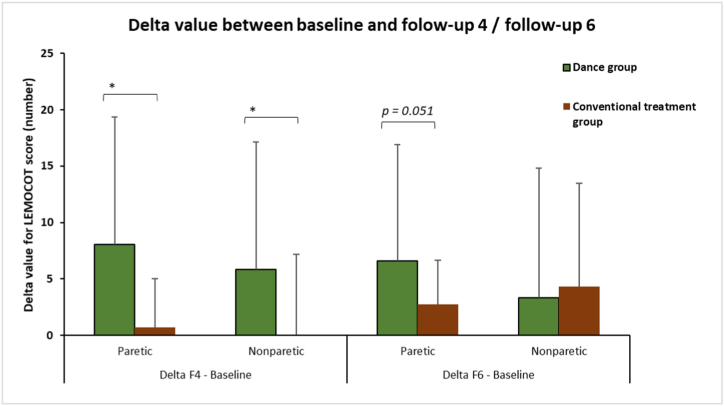
LEMOCOT results for delta value between baseline and follow-up 4 (F4) and follow-up 6 (F6) for dance group (green) and conventional treatment group (brown). The significant difference is represented by “*”.

The improvement in the LEMOCOT score was not significant for either group (Table 3). Nevertheless, the change values for paretic and nonparetic sides were significantly superior in the dance group than in the conventional treatment group at follow-up 4 (*p* ≤ 0.041; Fig. 4).

**Table 3:** Results at baseline, follow-up 4 (F4) and follow-up 6 (F6) for LEMOCOT.

Group	Side	Baseline	Follow-up 4	Follow-up 6	Diff (F4 – baseline)	Diff (F6 – baseline)	*p value* F4 vs. baseline	*p value* F6 vs. baseline
Dance	Paretic	20.53 ± 14.75	28.56 ± 11.95	27.12 ± 11.53	8.03 ± 11.36	6.59 ± 10.35	0.109	0.109
	Non Paretic	33.90 ± 17.35	39.75 ± 11.08	37.25 ± 10.70	5.84 ± 11.30	3.34 ± 11.46	0.233	0.460
Conventional treatment	Paretic	7.87 ± 10.91	8.56 ± 12.82	10.62 ± 13.49	0.68 ± 4.36	2.75 ± 3.92	0.674	0.105
	Non Paretic	29.12 ± 15.26	28.06 ± 15.33	33.43 ± 19.01	−1.06 ± 8.23	4.31 ± 9.19	0.742	0.141
*p value* between groups	Paretic	***0.022***	***0.004***	***0.011***	***0.015***	0.051		
	Non Paretic	0.299	0.063	0.356	***0.041***	0.479		

The *p* value in italic and bold represents a significant result (*p < 0.050*).

Measuring the knee extensor muscle strength revealed no significant differences between the dance group (paretic side: 25.01 ± 12.22 % baseline, 25.89 ± 14.35 % follow-up 4, and 28.94 ± 14.78 % follow-up 6; nonparetic side: 34.05 ± 7.55 % baseline, 34.35 ± 12.96 % follow-up 4, and 35.83 ± 12.22 % follow-up 6; *p ≥* 0.195) and the conventional treatment group (paretic side: 17.50 ± 12.96 % baseline, 18.84 ± 17.26 % follow-up 4, and 19.12 ± 16.23 % follow-up 6; nonparetic side: 28.12 ± 9.20 % baseline, 29.32 ± 13.11 % follow-up 4, and 29.81 ± 8.76 % follow-up 6; *p* ≥ 0.352). The comparison between groups was nonsignificant for both sides and for all three test times.

For the cognition (MoCA) and quality of life (SS-QOL) scores, no significant differences were observed between testing sessions and between groups (*p* > 0.050; Table 4).

**Table 4:** Results for SS-QOL and MoCa for dance and control groups.

	SS-QOL (score/245)					MoCA				
	Baseline	Follow-up 4	Follow-up 6	*p value* F4 vs. baseline	*p value* F6 vs. baseline	Baseline	Follow-up 4	Follow-up 6	*p value* F4 vs. baseline	*p value* F6 vs. baseline
Dance group	147.62 ± 20.47	162.5 ± 19.48	167 ± 21.51	0.796	0.781	22.56 ± 5.23	22.37 ± 6.43	22.37 ± 6.82	0.778	0.725
Conventional treatment group	141.62 ± 39.67	153.62 ± 51.71	155.50 ± 40.31	0.271	0.262	20.12 ± 5.45	20.37 ± 5.42	20.25 ± 4.62	0.731	0.832
*pvalue* between groups	0.437	0.437	0.215			0.146	0.185	0.171		

The dance satisfaction questionnaire revealed that the joy of participation and improvement of balance and coordination were the greatest benefits of dance practice (Table 5). The majority of the participants wished to continue this activity after leaving the rehabilitation center.

**Table 5:** Results of dance satisfaction.

Score/5 (1 = strongly disagree; 5 = strongly agree)	I enjoyed participating in this dance program	My balance has improved	My walking has improved	My mood has improved	My coordination has improved	My strength has improved	My endurance has improved	If I could, I would continue participating	I have been more physically active	Total (/45)
Mean	4.25	3.5	2.75	4	3.5	2.75	2.75	3.75	3	30.25
Median	4.5	4	2.5	4	4	3	3	4.5	3	31
Min	3	1	1	3	1	1	1	1	1	18
Max	5	5	5	5	5	4	4	5	5	41

## 4 Discussion

The aim of the study was to assess how a dance class affected people's motor recovery during the subacute phase following a stroke. Given the early termination of the study, which prevented the sample size target from being met, the results obtained should be carefully interpreted as those of a pilot study. Based on the results, one dance class per week combined with standard rehabilitation improved motor capacities, functional independence, and coordination recovery. On the other hand, there was no difference in quality of life, muscle strength, or cognitive function between the groups. Dance was regarded by the participants as an activity that improved balance, coordination, and joy. Therefore, employing this artistic activity during the subacute phase appears to be a particularly relevant approach to supporting post-stroke rehabilitation for functional recovery. The COVID-19 health crisis forced the study's termination, so these results should be interpreted cautiously.

The participants demonstrated excellent tolerance for the duration and intensity of the dance class, confirming its excellent feasibility for stroke survivors, even during the subacute phase [13,14,38]. One of the unique characteristics of this innovative approach in this neurological context is the participants’ positive perception of its physical, mental, and social benefits [39] even with only one session per week. This excellent self-perception had a positive impact on the motivation of stroke survivors, which may partly explain the positive effects on motor recovery in the subacute phase [38]. As in the chronic phase [15,16], the influence of dance practice in the subacute phase was primarily evident on postural control [18], with greater improvement than in patients who underwent conventional rehabilitation alone. Compared to conventional treatment, dance has the advantage of providing varied movement sequences, including changes in direction, rhythm, and spatiotemporal characteristics, as well as choreography with synchronization between body parts, which can be crucial for enhancing dynamic balance [15]. The Mini-BESTest includes dynamic and static balance items that seem particularly suited to identifying balance recovery in the case of dance intervention. These varied balance abilities are essential for managing postural control in everyday activities [6]. Moreover, as was previously demonstrated for dance classes for adults with cerebral palsy [40], participants in the dance group appear to have improved their balance and confidence. Actually, there was no significant difference between the groups; however, the follow-up score showed a significant improvement only for the dance group. If confidence in the ability to maintain balance is improved, individuals may be more likely to move outside of rehabilitation sessions, which may be advantageous to their recovery. However, dance is a complex activity that requires spatial orientation, rhythm ability, and synchronization between external stimuli and the whole body to produce artistic movement patterns [18]. This combination of cognitive, motor, and social stimulation could be the key to improving coordination and functional independence [6]. After 6 weeks of dance intervention, an improvement in the FIM measure was observed, whereas the LEMOCOT results were unclear. In patients with hemiparesis, the LEMOCOT and FIM are not always assessed, but in the subacute phase, these tests are a relevant indicator of motor recovery [31,41]. Following a stroke, the FIM, which measures independence in daily activities, is improved by aerobic-type exercises (40 –50 % heart rate recovery progressing to 60 –80 %) [42], such as dancing.

Despite the fact that dance offers a multimodal approach, including physical, psychological, and social components [14], no improvement was observed in cognition or quality of life. Teixeira-Machado et al. (2019) [43] have demonstrated that studies observing an improvement in cognition following a dance program were conducted on healthy or older individuals, whereas the findings for cognitive pathologies were more inconsistent, possibly due to the low intensity and diversity of dance sessions. A meta-analysis in the post-stroke context demonstrated the efficacy of combining cognitive and exercise interventions, but at a frequency higher than three times per week during the subacute stage [44], which might suggest that the number of dance sessions was too low to observe any improvement. The effect of dance on cognitive abilities after a stroke has not been researched before. Further investigation is needed. Regarding quality of life, the SS-QOL has already been used in the subacute phase [45]; however, several items are not suitable for patients hospitalized in rehabilitation centers (e.g., work/productivity and social and family roles). As a result, measurement may have limited the scale's sensitivity.

After a stroke, aerobic exercise is essential to decrease infarct volume and muscle fatigue and increase brain activity during movement, postural control, and coordination [46]. Mang et al. (2013) demonstrated that these aerobic exercises promote neuroplasticity through direct effects (e.g., the increase of neurotrophic growth factors, neurogenesis, neuroprotection, and neurotransmitters) and indirect effects (e.g., physical fitness improvements and the decrease of central nervous system inflammation) [43]. Therefore, aerobic activities, such as dancing, are recommended early on in stroke rehabilitation to improve brain health and promote cognitive and motor recovery [43]. Previous studies have demonstrated that dancing is particularly suitable for promoting neuroplasticity [43,47]. This activity involving repeated, varied exercises with different sensory inputs appears to optimize neuroplasticity in older adults compared to standard repeated exercises [48]. Although the effects of dance on neuroplasticity after a stroke have not been studied directly, this activity appears to meet many of the recommendations for exercises to optimize cognitive-motor recovery (i.e., cognitive-motor stimulation, an enriched environment, motivational factors, and social interaction) [8]. The integration of dance as a complement to conventional rehabilitation appears to be optimal for promoting the potural control and motor recovery of stroke survivors while offering an activity that provides pleasure in movement and social interaction. For cognitive abilities, the proposed frequency seems insufficient to obtain results. Future research must determine whether other dance modalities (e.g., number of sessions per week and other course content) would have a beneficial effect.

In addition to recovery-related considerations, offering physical activities during the subacute phase designed for each person to have an active lifestyle despite motor and cognitive limitations is crucial. Stroke survivors walk less than half as much as people their age on a daily average, even though exercise improves social, mental, and physical health as well as functional independence [49]. Moreover, physical activities practiced in groups promote autonomy and socialization [50]. Given the interest in cultural activities expressed by stroke survivors [19], dance appears to be an excellent activity that could also motivate individuals who are less responsive to traditional activities such as cycling and walking.

### *Limitations*

The main limitation was the small sample size, which resulted from the early termination of the randomized controlled trial due to the COVID-19 health crisis. Nevertheless, we used the guidelines to report a terminated trial [51]. Despite the small sample size, we followed to the RCT method and observed significant results using appropriate statistical nonparametric tests. A second limitation was the number of dance sessions per week. For reasons of convenience and organization in the neurorehabilitation department, only one session per week was proposed, although previous stroke studies have generally recommended two sessions per week [13]. This choice was approved because positive results had previously been demonstrated with patients suffering from other pathologies [25,26]. Compared to Demers et al.’s study from 2015 [18], the participants in our research had one fewer dance session per week, and the duration was 6 weeks instead of 4 weeks. Finally, at baseline, the dance group was younger than the conventional treatment group, and the Chedoke-McMaster score was higher only for the paretic foot, with no significant difference in any other parameters. Several studies have demonstrated that age is a predictor of recovery, but primarily for older individuals, particularly those over the age of 80 [52]. In our study, participants in both groups were on average under the age of 65 years, with those over the age of 80 being excluded. Regarding the outcomes, despite the nonsignificant difference at baseline, the Mini-BESTest, FIM, and ABC scale values were better for the dance group than for the conventional treatment group. These differences at baseline could potentially affect post-intervention results. We also observed a large standard deviation in the LEMOCOT results, which seem to demonstrate significant interindividual variation, as is frequently the case in studies of stroke survivors. Dancing may be intimidating to people who have never taken a dance class before [18]; however, after the first class, we observed that participants were often very enthusiastic and motivated.

## 5 CONCLUSION

Dance is an easy activity to implement in a neurorehabilitation center because it requires minimal equipment and a short amount of time. Although only one session per week was provided, this RCT demonstrated an improvement in the recovery of balance, coordination, and functional independence but no significant difference in quadriceps strength, cognitive capacities, or quality of life. The results of this study should be treated cautiously as they are from a pilot study, as the investigation has been stopped. Dance is greatly valued by the patients, and their perceptions of its benefits facilitate their participation. Subsequent research endeavors ought to validate these findings with a more extensive cohort and ascertain the optimal modalities for poststroke recuperation (e.g., weekly sessions, dance style, and length of instruction).

## Declarations section

### Ethics statement

This is a clinical trial including human subjects. Ethics approval has been granted by the Swiss Ethics Committee of the CER Vaud (2019-01467). In this article, there are no images of the participants. Authors comply with all relevant ethical regulations. Written consent was obtained for all participants.

## Data availability statement

Data are not available into a publicly available repository. Data will be made available on request.

## Trial registration number

NCT04120467.

Funding details.

This research is funded by an internal grant from the School of Health Sciences, Switzerland 10.13039/501100010743HES-SO//University of Applied Sciences and Arts Western Switzerland, Geneva, Switzerland and a generous patron advised by CARIGEST SA, Switzerland.

## Authors’ contributions

TP: performed experiment;

AR: performed experiment; contributed reagents, materials, analysis tools or data; wrote the paper.

## CRediT authorship contribution statement

**Anne-Violette Bruyneel:** Writing – review & editing, Writing – original draft, Visualization, Validation, Supervision, Resources, Project administration, Methodology, Funding acquisition, Formal analysis, Data curation, Conceptualization. **Thomas Pourchet:** Investigation. **Aline Reinmann:** Writing – review & editing, Writing – original draft, Resources, Investigation, Formal analysis.

## Declaration of competing interest

The authors declare the following financial interests/personal relationships which may be considered as potential competing interests.
