# Supplementary material for: Dance after stroke improves motor recovery in the subacute phase: A randomized controlled trial
Source: Heliyon. 2023 Nov 13;9(11):e22275. doi: 10.1016/j.heliyon.2023.e22275 (PMC10694307; doi:10.1016/j.heliyon.2023.e22275)
Supplement: Multimedia component 1 [file mmc1.pdf]

1. **Functinal Independance Measure (french)**

Score total : ____________

Activité	Score	Précisions *(à entourer)*
**Soins Personnels**		
Alimentation	/7	
Soins de la présentation et de l'apparence	/7	
Toilette (se laver)	/7	
Habillage haut du corps	/7	
Habillage bas du corps	/7	
Utilisation des toilettes (hygiène)	/7	
**Contrôle sphincters**		
Contrôle de la vessie	/7	
Contrôle des selles	/7	
**Transferts**		
Transferts lit, chaise, fauteuil roulant	/7	
Transferts aux toilettes	/7	
Transferts baignoire, douche	/7	
**Déambulation**		
Marche, Fauteuil roulant	/7	Marche, fauteuil roulant, les 2
Escaliers	/7	
**Communication**		
Compréhension	/7	Auditive, visuelle, les 2
Expression	/7	Verbal, non-verbal, les 2
**Capacités relationnelles et cognitives**		
Interaction sociale	/7	
Résolution des problèmes de la vie quotidienne	/7	
Mémoire	/7	

Hsueh I-P, Lin J-H, Jeng J-S, Hsieh C-L. Comparison of the psychometric characteristics of the functional independence measure, 5 item Barthel index, and 10 item Barthel index in patients with stroke. J Neurol Neurosurg Psychiatry. 2002 August;73:188–190.

2. ABC-scale (french)

Score: ____ /45 ⇨ ______%

Nom : ______________________________ Dossier :________________

Établissement :___________________ Évaluateur : _________________ Date : __________________

Jusqu'à quel point êtes-vous confiant(e) de garder votre équilibre lorsque vous faites les activités suivantes ?

ACTIVITÉS	Très confiant(e)	Moyen. confiant(e)	Un peu confiant(e)	Pas du tout confiant(e)
Vous balayez le plancher	3	2	1	0
Vous sortez de la maison pour aller vers une auto stationnée dans l'entrée	3	2	1	0
Vous vous étirez pour prendre une petite boîte de conserve sur une étagère, à la hauteur des yeux	3	2	1	0
Vous marchez dans la maison	3	2	1	0
Vous utilisez un escalier roulant en tenant la rampe	3	2	1	0
Vous traversez un terrain de stationnement pour vous rendre au centre commercial	3	2	1	0
Vous montez ou descendez de l'auto (régulière)	3	2	1	0
Vous marchez dans le centre commercial bondé de gens pressés	3	2	1	0
Vous vous penchez pour ramasser une pantoufle, sur le plancher de votre garde-robe	3	2	1	0
Vous montez ou descendez un plan incliné (rampe d'accès)	3	2	1	0
Vous montez ou descendez les escaliers	3	2	1	0
Vous êtes bousculé(e) par des gens en marchant dans le centre commercial	3	2	1	0
Vous vous tenez sur la pointe des pieds pour aller chercher un objet, au-dessus de votre tête	3	2	1	0
Vous êtes monté(e) sur une chaise (ou un escabeau) pour aller chercher un objet	3	2	1	0
Vous utilisez un escalier roulant sans pouvoir tenir la rampe parce que vous avez les bras chargés de paquets	3	2	1	0

© 2007 - Équipe d'évaluation du programme PIED (version traduite et simplifiée : l'échelle ABC-S)
© 1995 - Powell et Myers (version originale : le ABC Scale)

Cleary K, Skornyakov E. Predicting falls in community dwelling older adults using the Activities-specific Balance Confidence Scale. Arch Gerontol Geriatr. 2017 September;72:142–145.

3. SS-QOL (french)

Legris N, Devilliers H, Daumas A, Carnet D, Charpy J-P, Bastable P, Giroud M, Béjot Y. French validation of the Stroke Specific Quality of Life Scale (SS-QoL). NeuroRehabilitation. 2018;42:17–27.

Score

1 - Aide totale - N'a pas pu le faire du tout - Tout à fait d'accord

2 - Beaucoup d'aide - Beaucoup de problèmes - Modérément d'accord

3 - Un peu d'aide - Des ennuis - Ni d'accord ni en désaccord

4 - Un peu d'aide - Un peu d'ennuis - Modérément en désaccord

5- Pas besoin d'aide - Pas de problème du tout - Fortement en désaccord

Energie

1. Je sens fatigué la plupart du temps. ____

2. Je dois m'arrêter et me reposer pendant la journée. ____

3. Je suis trop fatigué pour faire ce que je voulais faire. ____

Rôles familiaux

1. Je ne participe pas à des activités uniquement pour m'amuser avec ma famille. ____

2. Je me sens un fardeau pour ma famille. ____

3. Ma condition physique perturbe ma vie personnelle. ____

Langage

1. Vous avez du mal à parler ? Par exemple, être coincé, butter dans les mots, bafouiller vos mots ? ____

2. Avez-vous de la difficulté à parler assez clairement pour utiliser le téléphone ? ____

3. D'autres personnes ont-elles de la difficulté à comprendre ce que vous avez dit ? ____

4. Avez-vous du mal à trouver le mot que vous vouliez dire ? ____

5. Devez-vous vous répéter pour que les autres puissent vous comprendre ? ____

Mobilité

1. Avez-vous de la difficulté à marcher (si le patient ne peut pas marcher, passez à la question 4 et notez les questions 2-3 comme ceci 1.) ____

2. Perdez-vous l'équilibre en vous penchant vers quelque chose ou en vous penchant vers quelque chose ? ____

3. Avez-vous de la difficulté à monter les escaliers ? ____

4. Devez-vous vous arrêter et vous reposer plus que vous ne le souhaiteriez en marchant ou en utilisant un fauteuil roulant ? ____

5. Avez-vous eu du mal à vous tenir debout ? ____

6. Avez-vous du mal à vous lever d'une chaise ? ____

Humeur

1. Etes vous découragé par votre avenir. ____

2. Vous n'avez pas d'intérêt pour d'autres personnes ou activités. ____

3. Vous vous sentez isolé des autres. ____

4. Vous avez peu de confiance en vous ____

5. La nourriture vous intéresse pas. ____

Personnalité

1. Je suis irritable. ____

2. J'ai été hospitalisé avec d'autres. ____

3. Ma personnalité a changé. ____

Soin de soin

1. Avez-vous besoin d'aide pour préparer la nourriture ? ____

2. Avez-vous besoin d'aide pour manger ? Par exemple, couper de la nourriture ou préparer de la nourriture ? ____

3. Avez-vous besoin d'aide pour vous habiller ? Par exemple, mettre des chaussettes ou des chaussures, boutonner des boutons, ou fermeture éclair ? ____

4. Avez-vous besoin d'aide pour prendre un bain ou une douche ? ____

5. Avez-vous besoin d'aide pour aller aux toilettes ? ____

Rôles sociaux

1. Je ne sors pas aussi souvent que je le souhaite. ____

2. Je fais mes passe-temps et mes loisirs pendant des périodes plus courtes que je ne le souhaite. ____

3. Je ne vois pas autant de mes amis que je le souhaite. ____

4. Je fais l'amour moins souvent que je ne le voudrais. ____

5. Ma condition physique perturbe ma vie sociale. ____

Pensées

1. J'ai du mal à me concentrer. ____

2. J'ai du mal à me souvenir des choses. ____

3. Je dois écrire des choses pour m'en souvenir. ____

Fonction des membres supérieurs

1. Avez-vous de la difficulté à écrire ou à dactylographier ? ____

2. Avez-vous du mal à mettre des chaussettes ? ____

3. Avez-vous du mal à boutonner les boutons ? ____

4. Avez-vous du mal à fermer une fermeture éclair ? ____

5. Avez-vous du mal à ouvrir un pot ? ____

Vision

1. Avez-vous de la difficulté à voir la télévision assez bien pour profiter d'un spectacle ? ____

2. Avez-vous de la difficulté à atteindre les choses à cause d'une mauvaise vue ? ____

3. Avez-vous du mal à voir les choses d'un côté ? ____

Travail / productivité

1. Avez-vous de la difficulté à faire le travail quotidien à la maison ? ____

2. Avez-vous de la difficulté à terminer les emplois que vous avez commencés ? ____

3. Avez-vous de la difficulté à faire le travail que vous faisiez auparavant ? ____

Reference

Williams LS, Weinberger M, Harris LE, Clark DO, Biller J. Development of a stroke-speci!c quality of life scale. Stroke 1999 Jul;30(7):1362-9.

4. MoCa (french)

Nom : | Date de naissance :
Scolarité : | DATE :
Sexe :

	Points
**VISUOSPATIAL/ÉXÉCUTIF**	
Copier le cube — *Dessiner l'horloge (onze heures dix)* 3 points	

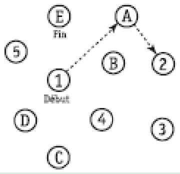

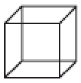

	Points
☐ ☐ ☐ ☐ ☐	☐/5
**DÉNOMINATION**	

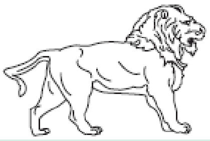
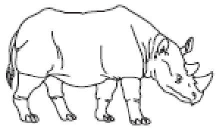
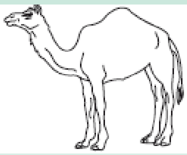

						Points
☐		☐		☐		☐/3
**MÉMOIRE** *Lire la liste de mots, le patient doit répéter. Faire un rappel 5 minutes après.*						
	**Visage**	**Velour**	**Église**	**Marguerite**	**Rouge**	**Pas de point**
**1er essai**						
**2e essai**						

	Points
**ATTENTION** *Lire la série de chiffres (1 chiffre/seconde)*	
Le patient doit la répéter ☐ 2 1 8 5 4	
Le patient doit la répéter à l'envers ☐ 7 4 2	☐/2
Lire la série de lettres. Le patient doit taper de la main à chaque lettre A. (Pas de point si 2 erreurs)	
☐ F B A C M N A A J K L B A F A K D E A A A J A M O F A A B	☐/1
Soustraire série de 7 à partir de 100 (4 ou 5 soustractions correctes : 3 pts ; 2 ou 3 correctes : 2 pts ; 1 correcte : 1 pt ; 0 correcte : 0 pt)	
☐ 93 ☐ 86 ☐ 79 ☐ 72 ☐ 65	☐/3
**LANGAGE** *Répéter :*	
"Le colibri a déposé ses œufs sur la table." ☐ "L'argument de l'avocat les a convaincus." ☐	☐/2
Fluidité du langage :	
Nommer un maximum de mots commençant par la lettre "F" en 1 minute ☐ (Nb. 11 mots)	☐/1
**ABSTRACTION**	
Similitude en ex. : banane, orange = fruit	
train – bicyclette ☐ *montre – règle* ☐	☐/2

**RAPPEL** (points pour rappel SANS INDICES seulement)						Points
Doit se souvenir des mots	*Visage*	*Velours*	*Église*	*Marguerite*	*Rouge*	☐/5
Sans indices	☐	☐	☐	☐	☐	
Indice de catégorie						
Indice choix multiples						

	Points
**ORIENTATION**	
☐ Date ☐ Mois ☐ Année ☐ Jour ☐ Endroit ☐ Vlle	☐/6
**TOTAL** (ajouter 1 point si scolarité ≥ 12 ans)	☐/30

Normal : 26/30

S m i t h T. e t a l . , 2 0 0 7 . w w w. m o c a t e s t . o r g

Toglia J, Fitzgerald KA, O'Dell MW, *et al*. The Mini-Mental state examination and Montreal cognitive assessment in persons with mild subacute stroke: relationship to functional outcome. *Arch Phys Med Rehabil* 2011;92:792–8.

5. **Satisfaction about dance program (french version)**

Merci de remplir le questionnaire le plus honnêtement possible.

1 = fortement en désaccord, 2 = plutôt en désaccord, 3 = ni en désaccord, ni en accord, 4 = plutôt d'accord et 5 = fortement d'accord

	fortement en désaccord	*plutôt en désaccord*	*ni en désaccord, ni en accord*	*plutôt d'accord*	*fortement d'accord*
Je suis content(e) de participer au programme de danse	1	2	3	4	5
Mon équilibre est amélioré	1	2	3	4	5
Ma marche est améliorée	1	2	3	4	5
Mon humeur est améliorée	1	2	3	4	5
Ma coordination est améliorée	1	2	3	4	5
Ma force est améliorée	1	2	3	4	5
Mon endurance est améliorée	1	2	3	4	5
Si c'est possible, je voudrais continuer à participer au cours	1	2	3	4	5
Je suis plus actif(ve) physiquement	1	2	3	4	5

Patterson KK, Wong JS, Nguyen T-U, Brooks D. A dance program to improve gait and balance in individuals with chronic stroke: a feasibility study. Top Stroke Rehabil. 2018;25:410–416.
